# Hepatic alveolar echinococcosis simulating metastatic malignancy

**DOI:** 10.4322/acr.2024.474

**Published:** 2024-02-08

**Authors:** Suvradeep Mitra, Payal Charaya, Shrinath Gururaj Deshpande, Mayur Parkhi, Thakur Deen Yadav

**Affiliations:** 1 Post Graduate Institute of Medical Education and Research, Department of Histopathology, Chandigarh, India; 2 Post Graduate Institute of Medical Education and Research, Department of GI Surgery, HPB, and Liver Transplantation, Chandigarh, India

**Keywords:** Keywords: Liver, Echinococcosis, Neoplasm Metastasis, Pathology

## Abstract

Echinococcosis is a parasitic disease caused by infection with tiny tapeworms of the genus *Echinococcus*. Echinococcosis is classified as either cystic echinococcosis or alveolar echinococcosis. The common form is a zoonosis from goats and sheep that tends to cause liver lesions. The larval stage of *Echinococcus multilocularis* causes alveolar echinococcosis/alveolar hydatid disease. It is a zoonosis with field mice and tundra voles as intermediate and wild carnivores like foxes and wolves as definitive hosts. This zoonosis is highly uncommon compared to the other form known as cystic echinococcosis but poses a great human threat if untreated. We report the case of a young man who was working in the Kashmir Valley, North India, and presented with jaundice and right upper quadrant abdominal pain. Computed tomography revealed a large solid-cystic intrahepatic lesion measuring 125x118x123 mm, suggestive of a malignant tumor with central necrosis. A liver biopsy showed necrosis with PAS-positive membranes morphologically consistent with echinococcosis. Alveolar echinococcosis can present as a solid-cystic mass in the liver and can simulate metastatic malignancy.

## INTRODUCTION

Alveolar echinococcosis is a zoonosis caused by *Echinococcus multilocularis*. It is an aggressive form of helminthic disease and often simulates disseminated metastatic malignancy. Echinococcosis is the differential diagnosis of various solid-cystic liver lesions in those parts of the world where echinococcosis is endemic. The diagnosis can be suspected in a patient with signs and symptoms of hepatic disease, history of exposure to the animal hosts, and typical radiological findings. The diagnosis can be confirmed on histopathology. We present a case of alveolar echinococcosis in a young man presenting with solid-cystic hepatic lesions clinically suspected to represent metastatic malignancy.

## CASE REPORT

**Figure 1:**
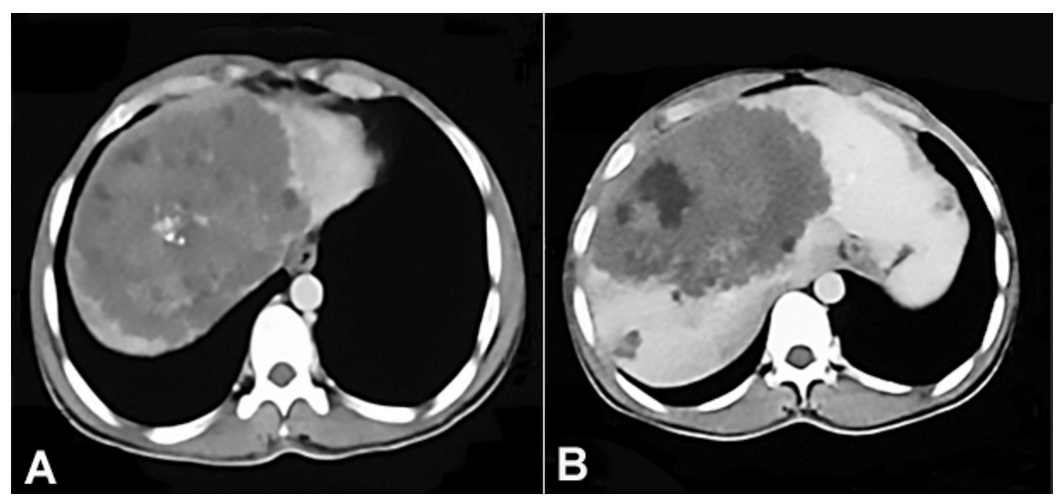
Contrast-enhanced computed tomography (CECT) revealing a large solid-cystic intrahepatic lesion with **A** - a few specks of calcification and **B** - central hypodense areas of necrosis.

A 36-year-old man presented to the outpatient department with jaundice for 1.5 months along with pain in the right upper quadrant for 1 week. There was no history of loss of appetite, weight loss, fever, or melena. He denied comorbidities and was a temporary resident of Kashmir, the northern most state of India, situated in the Himalayan foothills. On examination, the abdomen was soft and non-tender. A hard mass was palpable in the right upper quadrant. The abdominal contrast-enhanced computed tomography (CECT) revealed a large solid-cystic intrahepatic lesion measuring 125x118x123 mm involving the hepatic segments IV, VII, and VIII (Figure 1). 

This lesion showed peripheral subtle curvilinear and central nodular calcification. Arterial phase images revealed mild peripheral enhancement, while mild delayed phase enhancement was also noted. This lesion was found to compress the left portal vein and right main bile duct, causing right intrahepatic biliary radicle dilatation (IHBRD). Small cystic lesions were noted involving both lobes of the liver, the largest measuring 23x18 mm, and a few enlarged periportal lymph nodes (the largest measuring 18x14mm). The positron emission tomography and computed tomography (PET-CT) revealed peripherally FDG-avid necrotic lesions with central calcification. His peripheral blood investigations showed a mild eosinophil increase (9%) with normal hemoglobin and total leukocyte count. The liver function test revealed mild conjugated hyperbilirubinemia, mild transaminase elevation, elevated alkaline phosphatase, and a reversed albumin: globulin ratio (Table 1). His serum CEA, CA-19-9, and AFP levels were within normal limits.

**Table 1:** Results of various blood-based investigations in the patient

Parameters	Reference range	Results	
Hemoglobin	13.5-17.5 g/dL		13.2 g/dL
Total leukocyte	4000-11000/ µL		7390/µL
Platelet count	150,000-450,000/ µL		424,000/ µL
Absolut eosinophil count	665 cells/ µL		30-350 cells/ µL
Bilirubin (total)	0.2-1.2 mg/dL		1.61 mg/dL
Direct Bilirubin	0-0.3 mg/dL		1.32 mg/dL
AST	0-40 U/L		62.1 U/L
ALT	0-40 U/L		89.6 U/L
ALP	42-128 U/L		905 U/L
Total protein	6.4 - 8.3 g/dL		8.77 g/dL
Albumin	3.4 - 4.8 g/dL		3.16 g/dL

AST - aspartate aminotransferase; ALT - alanine aminotransferase; ALP - alkaline phosphatase.

**Figure 2:**
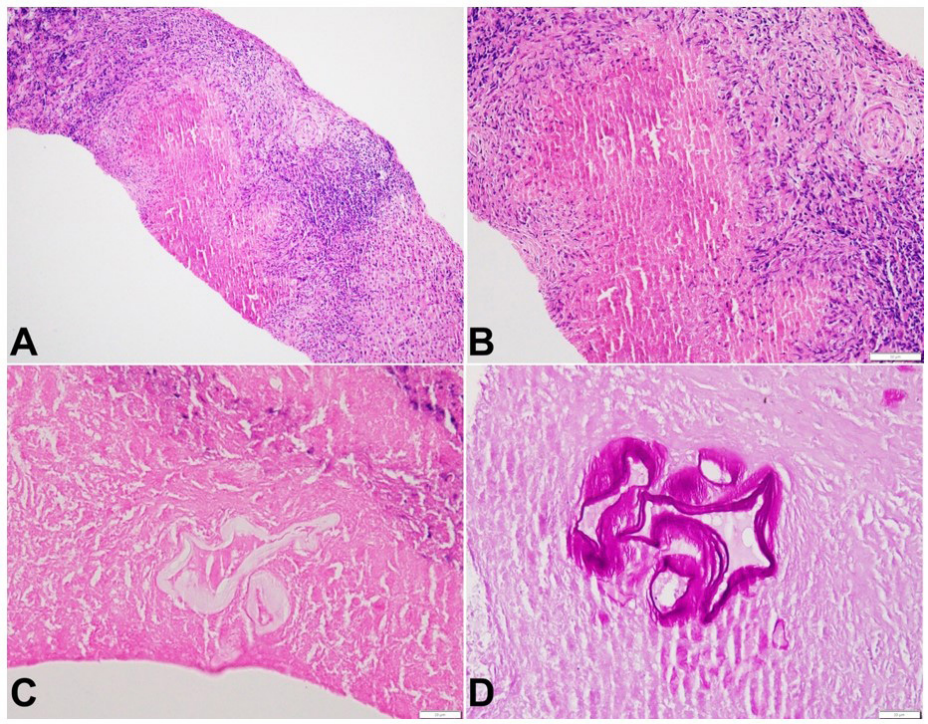
Photomicrographs of the liver biopsy. Histopathology of alveolar echinococcosis: **A** and **B** - Zones of eosinophilic necrosis surrounded by palisading histiocytes, fibrosis, and lymphocytic collar (H&E, A-100X, B-200X); **C** - refractile membranes at the center of necrosis (H&E, 400X); **D** - PAS-positive membranous structures (400X).

A clinical possibility of a multifocal/metastatic centrally necrotizing malignant mass versus alveolar hydatid disease was considered, and a liver biopsy was performed. The liver biopsy highlighted multiple randomly dispersed foci of eosinophilic necrosis surrounded by palisading histiocytes, a pale zone of fibrosis, and a thick collar of lymphocytes at places (Figures 2A and 2B). These foci of necrosis were dominantly lobular, at places confluent, giving rise to large necrotic areas. A few foci contained pale, refractile eosinophilic membranes, which were strongly PAS-positive (Figures 2C and 2D). No tumor was seen. This morphology was classical to alveolar echinococcosis.

The intervening portal tracts and the lobules showed variable degrees of inflammatory cell infiltration containing lymphocytes and occasional eosinophils. The individual is planned for surgical resection alongside treatment with anthelminthic agents.

## DISCUSSION

*Echinococcus multilocularis*, a tapeworm of the helminthic genus *Echinococcus*, causes alveolar echinococcosis/alveolar hydatid disease. It is a zoonosis with field mice and tundra voles as intermediate and wild carnivores like foxes and wolves as definitive hosts.^1,2^ Human beings serve as accidental intermediate hosts to this tapeworm due to the feco-oral transmission of the worm eggs.^1-4^ The infection occurs via ingesting wild berries/fruits or water contaminated with the fecal matter of the wild canids or via the handling of the wild animals.^1-4^ The infection is usually silent for many years, and the clinical manifestations generally develop after a long incubation period (5-15 years). Middle-East Asia, China, Siberia, and Alaska are the relatively commoner locations of AE, and a noteworthy proportion of cases of Alveolar echinococcosis from India either originate from Kashmir valley or have a travel history to the abovementioned countries.^1-3^ Indeed, the first two cases of AE documented from India belonged to the Uri region of the Kashmir valley.^1,3^ Kashmir valley is situated in the northern most part of India at the foothills of the Himalayas. It is rich in forest flora and fauna, suggesting higher chances of encountering wild canids or their excrement. Our patient, though not native, lived and worked in the Kashmir region.

Compared to the commonest form of hydatid disease caused by *E. granulosus*, *E. multilocularis* and *E. vogeli* are occasional. *E. multilocularis* infection is more aggressive, often lethal, and dominantly involves the liver. A few years back, such an unusual form of disease presentation at the cardiac site was documented in the literature.^5^ It often shows vascular and neural spread mimicking metastatic malignancies. Compared to *E. granulosus*, *E. multilocularis* does not have the typical exocyst, rendering the larvae to grow in direct contact with the tissue, perpetuating tissue necrosis and fibroinflammatory tissue response. The scolices are absent in Alveolar echinococcosis, and direct tissue-invasive growth of Alveolar echinococcosis can simulate malignancy.^3^ The differential diagnosis includes cystic neuroendocrine tumor, cystic metastatic deposits, biliary cystadenoma, mucinous cystic neoplasm, pyogenic abscess, amebic abscess, or cystic echinococcosis (CE). Ultrasonography (USG) of AE highlights a combination of both hyper and hypo-echogenic areas in a tumefactive lesion or a central area of necrosis (pseudocyst) surrounded by a hyperechogenic ring. World Health Organization - Informal Working Group on Echinococcosis (WHO-IWGE) proposes a PNM classification of AE based on the extent of the primary lesion in the liver (P), involvement of the neighboring organs (N), and metastatic organ involvement (M).^5^ The diagnostic criteria of AE as per the WHO-IWGE proposal require one of the four parameters, including 1. Typical radiology in typical organs, 2. Positive serology, 3. Compatible histopathology, and 4. Detection of *E. multilocularis* nucleic acid sequence in a clinical specimen.^6^ Our patient showed both typical radiology in the liver and compatible histopathology.

The histopathology of Alveolar echinococcosis is characteristic and is akin to this case. Identifying the PAS-bright lamellated membranes within areas of necrosis and/or fibrosis in the liver parenchyma remain at the crux of the diagnosis. The typical eosinophilic necrosis surrounded by palisading histiocytes and inflammatory cells with/without fibrosis provides the necessary clue to the diagnosis in many cases.

## CONCLUSION

Alveolar echinococcosis is an uncommon form of Echinococcus-related helminthic infection. It is often lethal, and mimics disseminated metastatic malignancy. Astute clinical history, accurate epidemiological evaluation, and timely radiological and/or histopathological diagnosis help in the correct management of the case and prevent unnecessary treatment complications.
